# Insight in cognitive impairment assessed with the Cognitive Assessment Interview in a large sample of patients with schizophrenia

**DOI:** 10.1007/s00406-023-01641-7

**Published:** 2023-06-28

**Authors:** Paola Bucci, Armida Mucci, Giulia M. Giordano, Edoardo Caporusso, Luigi Giuliani, Dino Gibertoni, Alessandro Rossi, Paola Rocca, Alessandro Bertolino, Silvana Galderisi, on the behalf of the Italian Network for Research on Psychoses, Giuseppe Piegari, Eleonora Merlotti, Francesco Brando, Marco Papalino, Vitalba Calia, Raffaella Romano, Stefano Barlati, Giacomo Deste, Paolo Valsecchi, Federica Pinna, Alice Lai, Silvia Lostia Di Santa Sofia, Maria Salvina Signorelli, Laura Fusar Poli, Teresa Surace, Giovanni Martinotti, Chiara Montemitro, Silvia Fatricelli, Mario Altamura, Eleonora Angelini, Antonella Elia, Pietro Calcagno, Martino Belvederi Murri, Simone Cattedra, Francesca Pacitti, Rodolfo Rossi, Valentina Socci, Laura Giusti, Anna Salza, Silvia Mammarella, Andrea de Bartolomeis, Angela Favaro, Enrico Collantoni, Paolo Meneguzzo, Matteo Tonna, Paolo Ossola, Maria Lidia Gerra, Carla Gramaglia, Valeria Binda, Eleonora Gambaro, Claudia Carmassi, Barbara Carpita, Ivan Mirko Cremone, Giulio Corrivetti, Giammarco Cascino, Gianfranco Del Buono, Roberto Brugnoli, Anna Comparelli, Valentina Corigliano, Antonio Buzzanca, Nicoletta Gerardi, Marianna Frascarelli, Andrea Fagiolini, Arianna Goracci, Simone Bolognesi, Alberto Siracusano, Giorgio Di Lorenzo, Michele Ribolsi, Cristiana Montemagni, Cecilia Riccardi, Elisa Del Favero

**Affiliations:** 1 Department of Psychiatry, University of Campania “Luigi Vanvitelli”, Naples, Italy; 2 Research and Innovation Unit, IRCCS Azienda Ospedaliero-Universitaria di Bologna, Bologna, Italy; 3 Section of Psychiatry, Department of Biotechnological and Applied Clinical Sciences, University of L’Aquila, L’Aquila, Italy; 4 Department of Neuroscience, Section of Psychiatry, University of Turin, Turin, Italy; 5 Department of Basic Medical Science, Neuroscience and Sense Organs, University of Bari ‘Aldo Moro’, Bari, Italy

**Keywords:** Schizophrenia, Cognitive impairment, Co-primary measure, Insight, Functioning

## Abstract

The Cognitive Assessment Interview (CAI) is an interview-based scale measuring cognitive impairment and its impact on functioning in subjects with schizophrenia (SCZ). The present study aimed at assessing, in a large sample of SCZ (*n* = 601), the agreement between patients and their informants on CAI ratings, to explore patients’ insight in their cognitive deficits and its relationships with clinical and functional indices. Agreement between patient- and informant-based ratings was assessed by the Gwet’s agreement coefficient. Predictors of insight in cognitive deficits were explored by stepwise multiple regression analyses. Patients reported lower severity of cognitive impairment vs. informants. A substantial to almost perfect agreement was observed between patients’ and informants’ ratings. Lower insight in cognitive deficits was associated to greater severity of neurocognitive impairment and positive symptoms, lower severity of depressive symptoms, and older age. Worse real-life functioning was associated to lower insight in cognitive deficit, worse neurocognitive performance, and worse functional capacity. Our findings indicate that the CAI is a valid co-primary measure with the interview to patients providing a reliable assessment of their cognitive deficits. In the absence of informants with good knowledge of the subject, the interview to the patient may represent a valid alternative.

## Introduction

An impairment of several neurocognitive domains has been widely reported in patients with schizophrenia and is regarded as a core feature of the disorder [1–6]. It can be observed before the onset of the disorder [7, 8], often persist after symptom remission, and during periods of clinical stability [9, 10] and has been found, though less severe, in unaffected first-degree relatives of patients with schizophrenia [10–12], thus representing a possible vulnerability factor for the disorder. According to the findings of a large body of literature, cognitive deficits are among the strongest predictors of functional outcome in subjects with schizophrenia, showing an even greater impact on social functioning than positive and negative symptoms [13–20].

For these reasons, cognition is increasingly considered an important target for schizophrenia treatment [21–23], and a reliable and feasible assessment of cognitive deficits represent a crucial point for the implementation of specific treatments and the assessment of their efficacy. To assess the cognitive domains more frequently impaired in schizophrenia, a comprehensive consensus cognitive battery, the NIMH-Measurement and Treatment Research to Improve Cognition in Schizophrenia (MATRICS) Consensus Cognitive Battery (MCCB) was developed [24, 25]. This is a performance-based instrument now regarded as the ‘state-of-the-art’ neuropsychological battery for research purposes in schizophrenia and other severe psychiatric disorders [26].

However, within the MATRICS initiative, the U.S. Food and Drug Administration (FDA) indicated the need to integrate primary measures of cognitive functioning, obtained using a standardized neuropsychological battery, with co-primary measures, such as interview-based evaluations [27, 28]. Within this initiative, the Cognitive Assessment Interview (CAI) [29] was developed; it is an interview-based measure of cognition aimed at measuring the subjective perception of the impact on functioning of the cognitive impairment.

Interview-based cognitive assessments may have several advantages: (a) they are easier to use in clinical contexts than neuropsychological test batteries; (b) can provide a self-evaluation of cognition by patients, as well as an evaluation by caregivers, which may increase motivation to adhere to cognitive rehabilitation programs and awareness of the impact of cognitive deficits on real-life functioning; (c) may enable the identification of subtle cognitive deficits in spite of a performance within normal range on neuropsychological tests; (d) may allow the identification of cognitive improvements induced by treatments which may be only subjectively perceived [30–34].

Although the subjective assessment of cognition may provide valuable information, it may be influenced by some limitations. First of all, reliability of patients’ reports may be affected by the level of insight of the impact of their cognitive deficits on real-life functioning. Other potential limitations include reliability of informants’ reports which varies according to their characteristics, such as their level of knowledge of the patient functioning, as well as the difficulty in some contexts to find a caregiver available to be interviewed [28].

Clinical insight in schizophrenia is a complex construct which underwent changes in its definition in the last decades [35–37] and it is now regarded as a degree of awareness of the illness, rather than a binary concept [38]. A poor level of clinical insight into their condition and into the need of treatment is a common feature among patients with schizophrenia with respect to other mental disorders [38, 39]. However, clinical insight definition focuses on the degree of awareness of abnormal experiences, especially psychotic symptoms, such as delusions and hallucinations, or disorganization [35–38].

Whether the poor insight of patients suffering from schizophrenia extends to cognitive deficits is still a controversial issue. Studies investigating the convergence between patients’ subjective ratings and objective measures of cognitive performances provided non-conclusive findings. A review on this topic [40] highlighted that, among 26 included studies, approximately half found a good correspondence between subjective and objective measures of cognition, while the remaining half did not. Findings of further studies did not clarify the picture, as poor association between subjective and objective assessments was found in the majority of studies [28, 41–43] but not in all [34, 44]. Moreover, the lack of convergence between subjective and objective assessments in some studies was limited to some cognitive domains [41, 45, 46]. Discrepancies in these findings may be related to the heterogeneity of tools adopted for both interview-based and performance-based assessments of cognitive functioning and to the different cognitive domains considered in different studies, as well as to the fact that the majority of studies were not based on representative samples, often including a small sample of subjects or subjects with schizoaffective disorder. A few studies investigated the correspondence between objective measures of cognitive performance and interview-based ratings provided by informants (i.e., patients’ caregivers) and reported a good correlation between the two measures, although in some studies, this convergence was limited to some cognitive domains [41, 44, 47]. Thus, even if coprimary interview-based measures were developed and shown to be reliable and strongly associated with objective neuropsychological assessments of cognition and real-life functioning [48, 49], it is uncertain whether the patient can be the source of information when no caregiver or high contact clinician is available [50]. The systematic investigation of agreement among different sources is crucial to translate interview-based assessment into clinical practice. As a matter of fact, in several health care settings, caregivers having regular contact with patients in real-life situations are not available and clinicians or staff members very often have limited knowledge of patients’ competence in everyday life [50].

The present study was carried out in a large sample of community dwelling persons with schizophrenia within the activities of the Italian Network for Research on Psychoses (NIRP).Using the Italian version of the CAI [51], we investigated the agreement of clinician ratings based only on patients’ report of their cognitive impairment with those based on informants’ reports to explore whether patients can be a reliable source of information in the absence of available and/or informed caregivers; we also examined factors associated with patients’ insight in their cognitive deficits, to explore if it can be influenced by specific demographic and/or clinical characteristics; finally, we investigated the associations of cognitive indices with real-life functioning to explore whether patients’ awareness of cognitive impairment has an impact on functional outcome. We hypothesized that the CAI interviews carried out with patients—at least with chronic and clinically stable ones—may provide a reliable assessment of their cognitive deficits; we also expected that a reduced awareness of cognitive impairment may have an impact on functional outcome and may, therefore, represent a target of intervention in personalized treatment programs.

## Materials and methods

### Subjects

The present study was carried out in a large sample of community dwelling persons with schizophrenia within the activities of the Italian Network for Research on Psychoses (NIRP). We used the database relevant to the 4-year follow-up study [20, 52] since the CAI interview was not included in the baseline assessments.

Study participants were patients recruited among those consecutively seen at the outpatient units of 24 Italian university psychiatric clinics and/or mental health departments. All patients included in the baseline study [15] who agreed to participate in the follow-up were enrolled. Inclusion criteria were a diagnosis of schizophrenia confirmed with the Structured Clinical Interview for DSM-IV-Patient version (SCID-I-P) -and an age between 18 and 66 years. Exclusion criteria were: (a) history of head trauma with loss of consciousness in the last 4 years; (b) progressive cognitive deterioration possibly due to dementia or other neurological illness diagnosed in the last 4 years; (c) history of alcohol and/or substance abuse in the last 6 months; (d) current pregnancy or lactation; (e) inability to provide an informed consent; (f) treatment modifications (any change in the antipsychotic treatment, either dosage or compound) and/or hospitalization due to symptom exacerbation in the last 3 months to ensure clinical stability of the sample. All subjects signed a written informed consent to participate after receiving a comprehensive explanation of the study procedures and aims.

The study protocol was approved by the Ethics Committee and has been conducted in accordance with the principles of the Declaration of Helsinki (59th World Medical Association General Assembly; October 2008).

### Assessments

#### Psychopathology

The Positive and Negative Syndrome Scale (PANSS) was used to rate the severity of two psychopathological dimensions: ‘Disorganization’–assessed using the PANSS item P2, to avoid overlap with cognitive impairment [52]and ‘Positive symptoms’–calculated according to Wallwork et al. [53] by summing the scores for delusions, hallucinatory behavior, grandiosity, and unusual thought content.

Negative symptoms were assessed using the Brief Negative Symptom Scale (BNSS) [54, 55], an instrument designed to overcome the problem of heterogeneity of these symptoms. In fact, it allows the identification of two separate factors: the “Experiential domain”, consisting of anhedonia, asociality, and avolition, and the “Expressive deficit domain” including blunted affect and alogia.

Depressive symptoms were assessed by means of the Calgary Depression Scale for Schizophrenia (CDSS) [56]. It includes nine items (depression, hopelessness, self-depreciation, guilty ideas of reference, pathological guilt, morning depression, early wakening, suicide, observed depression), each rated from 0 (absent) to 3 (severe).

#### Neurocognition—Performance-based assessment

The Measurement and Treatment Research to Improve Cognition in Schizophrenia (MATRICS) Consensus Cognitive Battery (MCCB) [25, 57] was used for the performance-based neurocognitive assessment. It includes tests for the assessment of seven distinct cognitive domains: processing speed, attention/vigilance, working memory, verbal learning, visual learning, reasoning/problem solving, and social cognition. The latter domain was not used, since a thorough assessment of social cognition was included in this study, as described below. Standardized T-scores corrected for age and gender using Italian normative data [10] were calculated to the same measurement scale with a mean of 50 and SD of 10. The MCCB provides two composite score options: the overall and the neurocognitive composite scores, which respectively include and exclude the social cognition domain. We used the latter as index of neurocognition.

#### Neurocognition—interview-based assessment

The CAI [29] is a semi-structured interview developed by shortening and modifying the CGI-Cogs [30] and the SCoRS [33] scales. It includes ten items investigating six cognitive domains derived from the MCCB (speed of processing, attention/vigilance, working memory, verbal learning and memory, reasoning and problem solving, and social cognition). Each item is scored from 1 to 7, with higher scores indicating greater impairment. A “not applicable” score is assigned if the subject interrupts the interview or if not enough information is available. The clinician assigns a score rating the extent to which the cognitive dysfunction influences expected functioning in the workplace, school or in the social environment, avoiding to rate the influence on functioning of other symptoms of the disorder. The interview should be administered to the patient (patient interview) and to an informant, for instance a caregiver or someone who knows patient’s daily functioning (informant interview). Separate scores are obtained from the patient and informant interviews. Patient’s interview scores reflect the judgment of the clinician exclusively based on patient’s interview, while informant’s interview scores reflect the clinical judgment based on the informant’s interview. In addition, the clinician assigns for all the items a composite score, reflecting his/her expert judgement based on all available sources of information, combining that obtained by both interviews (patient and informant) and, when available, other sources (e.g., chart, or other sources of information). At the end of the interview, a score from 1 to 7 is rated on the global severity of cognitive impairment reflecting the patient’s overall cognitive impairment. Also for the global score, there are three separate ratings (one based on the patient interview, one on the informant interview, and one on the composite scores). In the present paper, we used the Italian version of the CAI [51] and we focused on the difference between patient and informant scores.

#### Social cognition

Social cognition was assessed by the Facial Emotion Identification Test (FEIT) [58] and The Awareness of Social Inference Test (TASIT) [59].

FEIT explores emotion perception. It consists in identifying the correct emotion (joy, anger, fear, disgust, surprise, sadness or neutral) represented in a specific photo.

TASIT is a theory of mind test consisting of seven scales (positive emotions, negative emotions, sincere, simple sarcasm, paradoxical sarcasm, sarcasm enriched, and lie), organized into three sections: emotion recognition, social inference-minimal, and social inference-enriched. The mean of standardized scores of FEIT and TASIT was used as a composite score in the present study.

#### Functional capacity

Functional capacity was evaluated by the brief version of the University of California San Diego (UCSD) Performance-based Skills Assessment (UPSA-B) [60], a performance-based instrument that assesses “financial skills” and “communication skills”. A total score, ranging from 0 (worst performance) to 100 (best performance), was obtained summing the two domains.

#### Real-life functioning

Real-life functioning was assessed using the Specific Level of Functioning Scale (SLOF) [61, 62], an instrument endorsed by the panel of experts involved in the Validation of Everyday Real-World Outcomes (VALERO) initiative. It explores different domains of functioning. This is based on key caregiver’s judgment on behavior and functioning of patients. The SLOF includes 43 items exploring 6 domains: physical efficiency, skills in self-care, interpersonal relationships, social acceptability, everyday life skills (e.g., shopping, using public transportation), and work skills. In the present study, we only analyzed the three SLOF domains showing moderate functional impairment (interpersonal relationships, everyday life skills, and work skills), as for the other domains, ceiling effects were observed and there was a reduced variability in patients’ scores. For all SLOF scales, higher scores correspond to better real-life functioning.

### Statistical analysis

Patient-informant agreement was calculated on all ten items of the CAI scale, and on the CAI global score. The mean scores of patient- and informant-based interviews and their difference were compared to assess the agreement, and tested using the following tests: the Wilcoxon matched-pair test of the null hypothesis of equality of means, Lin’s concordance correlation, the percentage agreement, the Gwet’s agreement coefficient (AC) and its related probabilistic benchmark interval. It is preferred to Cohen’s kappa family of coefficients because it has been found to be more robust and to be able to avoid the paradox of negative agreement [63–66]. Gwet’s AC are categorized into the classes of agreement provided by Landis and Koch (Landis and Koch, 1977) as slight (0.00 to 0.20), fair (0.21–0.40), moderate (0.41–0.60), substantial (0.61–0.80), and almost perfect (0.81–1.00) using a probabilistic assignment, that takes into account the variance of the estimate (Gwet, 2014). The ordinal weighting to Gwet’s AC calculation was applied to assign an increasing penalty as disagreement between scores increased along the item range.

To investigate factors associated with cognitive insight, stepwise multiple regression analyses were run in which patient-informant difference on the CAI global score was entered as dependent variables (higher scores for this index indicate better patients’ insight in cognitive deficits); independent variables included demographic characteristics (age, gender, education), psychopathological dimensions (positive, disorganization, Expressive deficit domain, Experiential domain and depression) as well as cognitive indices (MCCB neurocognitive composite score, social cognition composite score, functional capacity).

To investigate cognitive predictors of real-life functioning, separate stepwise multiple regressions were run, in which the three areas of real-life functioning were entered as dependent variables, while independent variables included the following cognitive indices: patient-informant difference on the CAI global score, MCCB neurocognitive composite score, and UPSA-B total score.

## Results

### Subjects

Six hundred and one patients with a diagnosis of schizophrenia according to the DSM-5 criteria were included in the study. They were 415 men and 186 women, had a mean age of 45.24 ± 10.40 years, and a mean education of 11.70 ± 3.4 years. Demographic and clinical characteristics of the experimental sample are reported in Table 1.

**Table 1:** Demographic, clinical, and functional characteristics of the experimental sample

	Total *N* = 601
Males (*N*, %)	415 (69.05%)
Age (years, mean ± SD)	45.24 ± 10.40
Education (years, mean ± SD)	11.70 ± 3.39
Duration of illness (years, mean ± SD)	21.27 ± 10.48
PANSS positive factor	8.463 ± 4.30
PANSS disorganization item (P2)	2.46 ± 1.44
BNSS experiential domain	12.10 ± 7.71
BNSS expressive deficit domain	18.60 ± 9.79
CDSS total score	3.27 ± 3.63
MCCB composite score	31.11 ± 12.80
UPSA-B total score	68.75 ± 23.97
Social cognition composite score	−0.03 ± 0.87
SLOF interpersonal relationships	22.13 ± 6.08
SLOF everyday life skills	45.04 ± 9.61
SLOF working skills	20.02 ± 6.13

*PANSS* Positive and Negative Syndrome Scale, *BNSS* Brief Negative Symptom Scale, *CDSS* Calgary Depression Scale for Schizophrenia, *MCCB* Measurement and Treatment Research to Improve Cognition in Schizophrenia (MATRICS) Consensus Cognitive Battery, *UPSA-B* University of California San Diego (UCSD) Performance-based Skills Assessment, *SLOF* Specific Levels of Functioning

### Agreement among patient-based and informant-based CAI ratings

Patient-based ratings on all CAI items and on global ratings were lower than informant-based ratings, indicating that patients reported a lower severity of cognitive impairment with respect to informants. These differences were statistically significant for the global ratings and for all CAI items, except for the first one (“Difficulty maintaining newly learned verbal information in mind for brief periods”) (Table 2).

**Table 2:** Patient and informer’s CAI scores, concordance, and interrater agreement

	Patient			Informer			Wilcoxon matched-pairs test		Lin’s concordance correlation coefficient		Interrater agreement		
	Obs	Mean	Std. Dev	Obs	Mean	Std. Dev	test	*p* value		Percent agreement		Gwet's AC	Probabilistic benchmark interval
CAI 1	601	2.73	1.44	586	2.77	1.52	−1.0	0.300	0.717	0.908		0.784	0.600–0.800
CAI 2	599	2.61	1.52	585	2.83	1.60	−5.4	< 0.001	0.779	0.918		0.808	0.600–0.800
CAI 3	601	2.92	1.42	584	3.19	1.49	−6.3	< 0.001	0.741	0.910		0.786	0.600–0.800
CAI 4	600	2.48	1.44	586	2.79	1.56	−7.1	< 0.001	0.755	0.917		0.809	0.600–0.800
CAI 5	601	2.53	1.36	586	2.72	1.48	−4.2	< 0.001	0.747	0.922		0.820	0.600–0.800
CAI 6	601	2.22	1.35	584	2.33	1.40	−2.8	0.005	0.757	0.926		0.841	0.800–1.000
CAI 7	600	2.64	1.52	585	3.08	1.64	−10.1	< 0.001	0.724	0.902		0.767	0.600–0.800
CAI 8	601	2.73	1.54	586	3.15	1.67	−9.5	< 0.001	0.747	0.911		0.785	0.600–0.800
CAI 9	601	2.60	1.43	585	2.96	1.49	−7.5	< 0.001	0.705	0.902		0.770	0.600–0.800
CAI 10	599	2.58	1.47	585	3.01	1.56	−8.8	< 0.001	0.658	0.886		0.730	0.600–0.800
CAI Global	598	3.00	1.26	579	3.31	1.38	−8.1	< 0.001	0.766	0.917		0.810	0.600–0.800

*CAI* Cognitive Assessment Interview

Gwet’s AC ranged from 0.73 to 0.82, indicating substantial to almost perfect agreement among patients and informants for all the CAI items and an almost perfect agreement for CAI global ratings (Table 2).

Lack of difference between patient and informant global ratings was observed in 51.38% of cases. A negative value as result of the difference between the two types of ratings – indicating a lower severity of cognitive impairment reported by patients with respect to informants – was observed in 35.81% of cases, while the opposite pattern (i.e., positive value indicating a greater severity of cognitive impairment reported by patients compared to informants) was observed in 12.81% of cases.

### Stepwise multiple regression analyses

Results of multiple regression analyses run to investigate factors associated with reduced insight in cognitive impairment are reported in Table 3. Greater neurocognitive impairment, greater severity of the positive psychopathological dimension, and older age were associated to lower insight in cognitive deficits; greater severity of depressive symptoms was associated to a better insight in cognitive impairment (Table 3).

**Table 3:** Stepwise multiple regression analysis on cognitive insight

Patient-informant difference on CAI global ratings		F(4485)		b p
	Age	8.27	−0.009	.004
	Gender			
	PANSS positive factor	6.40	−0.023	.01
	PANSS disorganization item (P2)			
	BNSS_experiential domain			
	BNSS expressive deficit domain			
	CDSS total score	6.10	0.031	.01
	MCCB composite score	21.48	0.012	.000005
	Social cognition composite score			
	UPSA-B total score			

*CAI* Cognitive assessment interview, *PANSS* Positive and Negative Syndrome Scale, *BNSS* Brief Negative Symptom Scale, *CDSS* Calgary Depression Scale for Schizophrenia, *MCCB* Measurement and Treatment Research to Improve Cognition in Schizophrenia (MATRICS) Consensus Cognitive Battery, *UPSA-B* University of California San Diego (UCSD) Performance-based Skills Assessment

Results of multiple regression analyses carried out to investigate cognitive predictors of real-life functioning are reported in Table 4. Greater impairment of neurocognition and functional capacity and lower insight in cognitive impairment were associated with worse functioning in all the three areas of real-life functioning. For the area, interpersonal relationships, the association with the neurocognitive composite score showed the highest level of statistical significance, followed by the patient-informant difference on CAI global score and by the UPSA-B total score; for everyday life skills, the association with the UPSA-B total score showed the highest level of statistical significance, followed by the patient-informant difference on CAI global score and by the neurocognitive composite score; for work skills, the association with the UPSA-B total score showed the highest level of statistical significance, followed by the neurocognitive composite score and by the patient-informant difference on CAI global score.

**Table 4:** Stepwise multiple regression analysis on real-life functioning

SLOF Interpersonal relationships		F(3486)		b p
	Patient-informant difference on CAI global ratings	7.69	0.825	.006
	UPSA-B total score	6.35	0.035	.01
	MCCB-neurocognitive composite score	28.97	0.061	< .000001
SLOF Everyday life skills		F(3486)	b p	
	Patient-informant difference on CAI global ratings	20.81	1.640	.000006
	UPSA-B total score	288.58	0.199	< .000001
	MCCB-neurocognitive composite score	11.16	0.111	.0009
SLOF Work skills		F(3486)	b p	
	Patient-informant difference on CAI global ratings	12.32	0.099	.0005
	UPSA-B total score	139.25	0.082	< .000001
	MCCB – neurocognitive composite score	21.83	0.099	.000004

*SLOF* Specific Levels of Functioning, *CAI* Cognitive Assessment Interview, *UPSA-B* University of California San Diego (UCSD) Performance-based Skills Assessment, *MCCB* Measurement and Treatment Research to Improve Cognition in Schizophrenia (MATRICS) Consensus Cognitive Battery

## Discussion

According to our findings, for all the CAI items, patients reported a lower impact of cognitive impairment on real-life functioning with respect to informants. This is in line with several previous studies reporting a tendency of patients with schizophrenia to overestimate their cognitive abilities with respect to those obtained by informants, by clinicians, and with neuropsychological batteries [4, 28, 46, 67–69]. However, we also found that patient ratings showed a substantial to almost perfect agreement with those of informants. In 51.38% of cases, patients did not underestimate the impact of their cognitive deficits on functioning. In the study by Gould et al. [68], that is the only other work in which the discrepancy of self-assessed vs. informant-rated CAI scores was investigated, in 40% of the cases, no underestimation of such an impact was found. The difference in the percentage of cases is probably related to the different methods of scoring and to the different informants used in the two studies. In fact, while, in our study, the ratings were assigned by the expert clinician interviewing the patient and the informant, who was a relative or a high contact staff member, in the study by Gould et al., the clinician assigned the ratings interviewing the patients and then rated the impairment of the patients based on his/her own appraisal of it. Notwithstanding the different methodology used in the two study, both Gould et al. and our own findings demonstrate that there is a substantial proportion of patients who present awareness of the impact that their cognitive deficits have on daily activities. Our findings of almost a perfect agreement between patient-based and informant-based ratings is of relevance to the field as not always it is possible to find informants who have a good knowledge of the patient functioning in real-life situations [47]. In our study, 64% of informants included a family member, either a relative or a partner, with a regular contact with the patient. This represents an advantage increasing reliability of informants with respect to what is described in other countries such as U.S., in which there is a lower percentage of family members among informants, with reduced contact with the patient [28, 47]. Thus, the agreement between patient-based and informant-based CAI ratings in our study lends support to the possibility of using the patients as the source of information for interview-based assessment of cognitive impairment in subjects living with schizophrenia.

The regression analyses carried out to investigate factors associated with the awareness of the impact of cognitive deficits on real-life functioning showed that lower awareness was associated to a greater severity of neurocognitive impairment and positive symptoms, lower severity of depressive symptoms and older age. The relationship between greater severity of positive symptoms and reduced insight has been reported in other studies [70, 71] and is an expected finding. However, this association was not found in some studies [67, 69] probably due to methodological factors such as the use of different types of cognitive interviews or differences in the characteristics of the experimental samples, such as lack of clinical stability, which may have an impact on the severity of positive symptoms. The positive association between depressive symptoms and subjective cognitive insight has been consistently reported in previous studies [28, 67, 69, 72] and may be related to the clinical characteristics of patients with depression [73]. It has been explained as a tendency of patients with depression to perceive a higher degree of cognitive impairment; this hypothesis is supported by the observation that depressive symptoms are correlated to subjective but not objective cognitive impairment [74]. In addition, depression has been related to a tendency to attribute negative experiences to internal causes; thus, it has been hypothesized that patients with depression may be excessively sensitive to normally occurring cognitive failure [42]. On the other hand, it cannot be excluded that a greater insight in subjective cognitive impairment contributes to the worsening of depressive symptoms.

On the whole, our findings on the level of agreement among patient-based and informant-based ratings, together with the above reported patterns of associations, suggest that the observed tendency to overestimate their cognitive abilities is limited to a proportion of patients characterized by the presence of more severe cognitive impairment and/or positive symptoms.

The regression analyses carried out to investigate cognitive predictors of real-life functioning showed that worse functioning on the three SLOF areas was associated to lower insight of the impact that cognitive deficits have on functioning, worse objective neurocognitive performance, and worse functional capacity. Our findings are in line with those of other studies on the relationships between insight and functioning [68]. In fact we confirmed that, in addition to the impairment in cognition and in functional capacity, that are well known predictors of functional outcome [14, 15, 17, 75], also the lack of awareness of cognitive impairment has an impact on real-life functioning and for some areas, the impact is even greater than the formers.

## Strengths and limitations

The main strengths of the present study are: (a) the large sample size including community dwelling subjects with schizophrenia; (b) the use of state-of-the-art instruments to conduct both performance-based and interview-based neurocognition assessments, as well as psychopathological and real-life functioning assessments.

The following study limitations have to be acknowledged: (a) lack of inclusion of the CAI in the baseline study, which did not allow to investigate its sensitivity to change over time; (b) lack of generalizability of findings to patients at their first episode of schizophrenia, or in acute phases of the illness, given the inclusion of chronic and clinically stable patients.

## Conclusion

In conclusion, our findings indicate that the CAI is a valid co-primary measure with the interview to the patients providing a reliable assessment of their awareness of the impact of cognitive deficits on functioning. In the absence of informants with high contact and good knowledge of the subject, the interview with the patient may represent a valid alternative.

The use of this interview in clinical practice to patients and caregivers may contribute to the implementation of comprehensive and personalized treatments, whose importance to improve outcome in schizophrenia has been highlighted in the recent literature [76–83] and may increase adherence to treatment by increasing awareness of cognitive dysfunction in patients and caregivers.

## Data Availability

The data that support the findings of this study are available from the corresponding author, GMG, upon reasonable request.
